# Revealing the impact of tadalafil-loaded proniosomal gel against dexamethasone-delayed wound healing via modulating oxido-inflammatory response and TGF-β/Macrophage activation pathway in rabbit model

**DOI:** 10.1371/journal.pone.0315673

**Published:** 2025-01-07

**Authors:** Nermin A. Helmy, Elsayed A. Abdel Aziz, Mustafa Abd El Raouf, Reda M. S. Korany, Doaa A. Mansour, Sara M. Baraka, Arwa A. Hassan, Eman Gomaa, Mennatullah M. Faisal, Walaa A. A. Basha, Esraa. M. Fahmy, Rashed A. Alhotan, Anam Ayyoub, Shaimaa Selim

**Affiliations:** 1 Department of Pharmacology, Faculty of Veterinary Medicine, Zagazig University, Zagazig, Egypt; 2 Department of Surgery, Anesthesiology and Radiology, Faculty of Veterinary Medicine, Zagazig University, Zagazig, Egypt; 3 Pathology Department, Faculty of Veterinary Medicine, Cairo University, Giza, Egypt; 4 Department of Biochemistry and Chemistry of Nutrition, Faculty of Veterinary Medicine, University of Sadat City, Sadat City Egypt; 5 Chemistry of Natural Compounds Department, National Research Centre, Giza, Egypt; 6 Pharmacology and Toxicology, Ministry of Health & Population, Cairo, Egypt; 7 Department of Pharmaceutics and Industrial Pharmacy, Faculty of Pharmacy, Zagazig University, Zagazig, Egypt; 8 Nanotechnology Research Center (NTRC), The British University in Egypt (BUE), Cairo, Egypt; 9 Anatomy and Embryology Department, Faculty of Veterinary Medicine, Suez Canal University, Ismailia, Egypt; 10 Department of Animal Production, College of Food and Agriculture Sciences, King Saud University, Riyadh, Saudi Arabia; 11 College of Life Sciences, Northwest A & F University, Xianyang, Yangling District, Shaanxi, China; 12 Department of Nutrition and Clinical Nutrition, Faculty of Veterinary Medicine, Menoufia University, Shibin El-Kom, Egypt; Dubai Pharmacy College, UNITED ARAB EMIRATES

## Abstract

A serious challenge of the chronic administration of dexamethasone (DEX) is a delay in wound healing. Thus, this study aimed to investigate the potential of Tadalafil (TAD)-loaded proniosomal gel to accelerate the healing process of skin wounds in DEX-challenged rabbits. Skin wounds were induced in 48 rabbits of 4 groups (n = 12 per group) and skin wounds were treated by sterile saline (control), TAD-loaded proniosomal gel topically on skin wound, DEX-injected rabbits, and DEX+TAD-loaded proniosomal gel for 4 weeks. The optical photography, transmission electron microscopy, in vitro release profile, and stability studies revealed the successful preparation of the selected formula with good stability. DEX administration was associated with uncontrolled oxido-inflammatory reactions, suppression in immune response in skin wounds, and consequently failure in the healing process. TAD-loaded proniosomal gel-treated rabbits manifested a marked enhancement in the rate of wound closure than control and DEX groups (p < 0.05). The TAD-loaded proniosomal gel successfully antagonized the impacts of DEX by dampening MDA production, and enhancing total antioxidant capacity, coupled with modulation of inflammatory-related genes, inducible nitric oxide synthase, tumor necrosis factor-alpha, interleukin-1β, and matrix metalloproteinase 9, during all healing stages (p < 0.05). This was in combination with significant amplification of immune response-related genes, CD68 and CD163 (p < 0.05). Moreover, the histopathological, Masson’s Trichrome-stain, and immune-histochemical studies indicated a successful tissue recovery with the formation of new blood vessels in groups treated with TAD-loaded proniosomal gel, as manifested by well-organized collagen fibers, upregulation of transforming growth factor β1, and vascular endothelial growth factor immune expression in skin tissues (p < 0.05). Overall, the topical application of TAD-loaded proniosomal gel is useful in improving the delayed wound healing linked to DEX therapy via regulating the release of inflammatory/macrophage activation mediators and enhanced antioxidant capacity, angiogenesis, and vascularity.

## 1. Introduction

The skin serves as a protective barrier between an individual and their surroundings. Any anatomical or physiological disturbance in the morphological structure of the skin that destroys cells and tissues of the skin is referred to as a wound. These injuries are becoming more dangerous for the world’s economy and health. Proper healing of wounds is crucial to the restoration of damaged functional status and anatomical stability of the skin [1, 2].

Wound healing is a dynamic process that incorporates four phases: hemostasis, inflammation, proliferation, and remodeling [3]. Hemostasis will begin as soon as an injury has occurred, resulting in blood clots made of fibrin and platelet cells [4]. Following the cessation of the bleeding, proinflammatory cytokines and growth factors are secreted [5]. After this phase comes the proliferative stage, marked by re-epithelialization‚ angiogenesis‚ and proliferation. The remodeling phase, in which the damaged area is restored by increasing the tensile strength [6].

Wound restoration is delayed when the stages of wound healing are out of balance. One of the most widely utilized glucocorticoids, dexamethasone (DEX), is employed as an immunosuppressive and anti-inflammatory agent to treat psoriasis and atopic dermatitis [7]. Regardless of these positive effects, DEX has been shown to have negative effects as well, including skin atrophy and slowing or impairing the wound healing process. This is because DEX suppresses the inflammatory and proliferation phases of this process [8], which is accomplished by reducing the proliferation of fibroblasts, synthesis of collagen triggered by the transforming growth factor β1 (TGF-β1) pathway, and decreasing turnover of the extracellular matrix (ECM) [9].

Because blood flow and vascular permeability are critical to the wound healing process, drugs that can impact these factors have been under research; phosphodiesterase type 5 (PDE-5) inhibitors are one example [10]. The commercially distributed PDE-5 inhibitors are vardenafil (Levitra), tadalafil (Cialis), udenafil (Zydena), and sildenafil (Viagra). Tadalafil (TAD) has lower adverse effects and a longer half-life time than other PDE-5 inhibitors since its clearance is primarily hepatic via the CYP3A enzyme, which has less of an impact on it, thus making it more beneficial for daily use [11]. Usually, TAD has been used for managing erectile dysfunction and pulmonary arterial hypertension by suppressing nitric oxide collapse-driven cGMP in platelets aggregation and vascular smooth muscle, which affects peripheral blood vessel vasodilation. Moreover, it protects against ischemia in various tissues, including the skin, brain, and lungs [12]. Also, TAD-loaded zein nanoparticles incorporated into pectin/PVA nanofibers had superior wound closure, skin regeneration, and collagen deposition in diabetic rat models [13].

Tadalafil cleaves the phosphodiester linkages in second messenger systems, such as cyclic guanosine monophosphate (cGMP), by acting as a competitive inhibitor of the PDE-5 enzyme. This increases cGMP, which amplifies nitric oxide’s (NO) vasodilatory action on endothelial and cellular levels [14, 15]. NO is a crucial signaling molecule that may speed up the healing of wounds by encouraging angiogenesis, tissue remodeling, and cell proliferation [16].

Since TAD is a poorly water-soluble drug [17], nanotechnology-based drug delivery systems such as proniosomes are recommended for improving lipophilic drugs’ stability and solubility and their bioavailability. Proniosomes are described as niosome concentrates with a gel-like texture because they contain less water; they are a better carrier for the transdermal and cutaneous delivery of many medicinal drugs. Proniosomes are more advantageous than niosomes and liposomes. For medications that are entrapped, proniosomes are less leaky than niosomes. Under occlusive conditions, they can convert into niosomes at the application site (such as the skin) [18, 19]. Consequently, in the current study, TAD was administered transdermally by encapsulating inside proniosomes using a coacervation phase separation process to improve skin penetration. TAD-loaded proniosomal gel was evaluated for its ability to enhance the wound healing process alone and in the presence of DEX-induced delayed wound healing in rabbits.

The objectives of the current trial were to determine the healing action of Tadalafil-loaded proniosomal gel against dexamethasone-induced delayed wound healing in a rabbit model via modulating oxido-inflammatory response and TGF-β/macrophage activation pathway.

## 2. Materials and methods

### 2.1. Drugs and chemicals

Dexamethasone ampules (Dexamethasone^®^) were purchased from Amria Pharmaceuticals Company (Cairo, Egypt), TAD powder was obtained from Mash Premiere Company (Cairo, Egypt), Span 20, Span 60, and cholesterol were obtained from Sigma Chemical Company (Sigma, St. Louis, MO, USA), Tween 80 was supplied from El-Nasr Pharmaceutical Chemicals Co. (Cairo, Egypt), Ethanol was acquired from El-Gomhouria Company for trading chemicals and medical appliances (Cairo, Egypt), and Lidocaine^®^ 2% was manufactured by Alexandria Company (Alexandria, Egypt).

### 2.2. Preparation of TAD-loaded proniosomal gel

#### 2.2.1. The preparation of proniosomal gels using the coacervation phase separation method

Tadalafil-loaded proniosomal gels were prepared in the lab of the pharmaceutics Department, Faculty of Pharmacy, Zagazig University (Zagazig, Egypt). They were prepared using, with some changes, the coacervation-phase separation process previously reported by El-Enin et al. [20]. Preliminary studies were conducted to determine the best concentration of each ingredient and the optimum processing conditions.

The ingredients of various proniosomal preparations are summarized in Table 1. Different amounts of multiple grades of the non-ionic surfactant (Span 20, Span 60, or Tween 80) were individually mixed with cholesterol (50 mg) and dissolved in 1 ml of absolute ethanol. Exactly 20 mg of the drug dissolved in 1 ml of acetone was added. The mixture was maintained at 65±3°C in a water bath until all of the components were dissolved. To each of the formed transparent solutions, 160μL of hot water was added while kept in the water bath for 3–5 min. This clear solution was later cooled and solidified into a proniosomal gel. For characterization, the formed gel was stored in the glass containers at 37°C. After 24hr duration, proniosomal gel formation was observed [20].

**Table 1:** Composition of different proniosomal formulations.

Formulation code	Span 20	Span 60	Tween 80	Cholesterol
F1		250		50
F2		150		50
F3	250			50
F4	150			50
F5			250	50
F6			150	50

#### 2.2.2. Characterization of proniosomal gel

*2*.*2*.*2*.*1*. *Particle size and zeta potential of TAD-loaded proniosomal gel*. An exact amount (100 mg) of the obtained proniosomal gel was weighed precisely and put into a tiny glass vial. The proniosomal gels were diluted with distilled water before particle size measurement. Three runs were performed to evaluate the average zeta potential and charge on the prepared proniosomal gel followed by hydration with phosphate buffer of pH (7.4) at 25 °C. The mean particle size and zeta potential of the formulations were measured using a zeta sizer instrument of the model (Malvern instruments, Nano ZS) according to the previous method reported by Reesha et al. [21].

The exact weight of 100 mg of the selected proniosomal gel was weighed and put in a small glass vial, the proniosomal gels were properly diluted with distilled water before particle size measurement. Three runs were conducted to evaluate the average zeta potential and charge on the prepared proniosomal gel followed by hydration with phosphate buffer of pH (7.4) at 25 °C. The mean particle size and zeta potential of the formulations were measured using a zeta sizer instrument of the model (Malvern instruments, Nano ZS) according to the previous method reported by Reesha et al. [21].

*2*.*2*.*2*.*2*. *Entrapment efficiency*. The entrapment efficiency of the prepared formulations was ascertained by separating the unentrapped drug using the centrifugation technique. Proniosomal gel was hydrated with a few drops of distilled water by hand shaking for 5 min to generate niosomal dispersion. Next, the mixture was centrifuged for 30 minutes at 25 °C at 15,000 rpm, then the obtained supernatant was used to measure the amount of free drug at λ_max_ of 284 nm using an ultraviolet (UV) spectrophotometer according to the previous method reported by Al-Okbi et al. [22] using the following equation:

$$\text{Entrapment}\;\text{efficiency}\left(\%\right)=\left[\left(\text{total}\;\text{drug-free}\;\text{drug}\right)/\left(\text{total}\;\text{drug}\right)\right]\times 100$$

*2*.*2*.*2*.*3*. *In vitro release study*. The specific weight of the generated proniosomal gel equivalent to 20 mg of TAD was transferred to a dialysis bag linked to the shaft of the dissolution equipment to measure the in vitro release of TAD from the prepared proniosomal gels using the membrane diffusion technique. The dissolution media was 100 mL of Sorensen’s phosphate buffer with pH 7.4 at a temperature of 37±0.5 °C and 50 rpm. At predefined intervals of time (1, 2, 4, 6, 12, and 24 hours); aliquots were removed and immediately refilled with the new buffer media. The drug content was then measured spectrophotometrically at λ_max_ of 284 nm three times for each sample c and the mean value ± SD was calculated [23].

*2*.*2*.*2*.*4*. *Determination of the pH and viscosity of the gel*. Using a viscometer and a spindle RV-3 is performed to assess the viscosity and the flow behavior of the prepared formulations. A digital pH meter with a glass electrode (Model 420, ORION, USA) was used to measure the pH of the prepared gel. After dissolving 0.1 g of gel in 10 mL of distilled water, the glass electrode was dipped into the gel formulation, and a continuous reading of the pH was recorded. An average of three readings were obtained [20].

*2*.*2*.*2*.*5*. *Investigation of gel surface morphology*. A drop of dispersed solution was placed on a glass slide without a cover slip to examine the surface morphology of the chosen proniosomal gel. Proniosomal formation was then investigated by using an optical microscope connected to a digital camera and high-resolution images were observed and captured [24].

*2*.*2*.*2*.*6*. *Transmission electron microscopy investigations*. Transmission electron microscopy (TEM; Model JEM-1230, JOEL, Tokyo, Japan) was used to investigate the morphology of the selected proniosomal formulation. A few drops of the formulation were placed on a carbon-coated grid and allowed to sit for two minutes to improve adsorption on the carbon film, excess liquid was removed by drying in the air using high-grade filter paper, and finally, a drop of (1%) phospho-tungstic acid was added as staining reagent of the sample [25].

The drug retention behavior was evaluated by storing the selected TAD-loaded proniosomal gel at 4 °C for 3 months. Proniosomal formulations were stored in glass vials covered with aluminum foil for the duration of the investigation. The stability analysis was conducted based on several metrics, such as physical characteristics, EE %, PS, and ZP by referring to the previous work reported by Moustafa et al. [26].

### 2.3. Biological investigations

#### 2.3.1. Experimental animals

Forty-eight New Zealand white rabbits (1.5–2.0 kg; aged 8 weeks) were acquired from the Experimental Animal House of the Faculty of Veterinary Medicine, Zagazig University, Egypt. The animals are housed individually in designated cages and fed a regular meal that includes an excess amount of water and fresh veggies. They were kept at 23 ± 2 °C with humidity of 45%–55% and on a 12-hour light-dark schedule. The procedure was carried out following the standards established by the Faculty of Veterinary Medicine Committee (Zagazig University, Egypt) on Research Ethics for Laboratory Animal Care (Approval Number, ZU-IACUC/2/F/395/2022).

#### 2.3.2. Wound induction

Rabbits were anesthetized with 2% Lidocaine hydrochloride and injected subcutaneously near the wound area (4.5 mg/kg) according to previous work reported by Valizadeh et al. [27]. Following anesthesia, the rabbits’ backs were cleaned and shaved, and a typical, round, full-thickness skin defect of 2.5 cm in diameter was created in the midline [28]. Upon recuperation, the animals were returned to their cages and given access to food and drink.

#### 2.3.3. Experimental design

Following the induction of a skin wound, the rabbits were split into 4 groups, with 12 rabbits per group as follows: the control group received sterile saline treatment for their skin wound, the TAD-group was treated topically with TAD-loaded proniosomal gel (20 mg/kg) [29], the DEX group was treated intramuscularly with DEX (2 mg/kg body weight, every 6 h) the day before the wound (at 6-hour intervals) and continued (2 mg/kg, every 48 h) during the treatment period [30], and the TAD + DEX group was treated by DEX, concurrently with TAD-loaded proniosomal gel locally to the wounded area.

The course of all therapies lasted for 28 days. On days 0, 7, 14, 21, and 28 of the trial, wounds were evaluated and photographically investigated. Four animals from each group were decapitated and their skin was removed on the 7th, 14th, and 21st days of the trial. For additional analysis, a portion of each animal’s skin was flash-frozen in liquid nitrogen and stored at -80°C. The remaining portion was preserved in 10% of the neutral formalin solution.

#### 2.3.4. Gross examination of wounds

The time the wound was created was day 0, and wounds were thoroughly examined every day for signs of infection or inflammation during the experiment. Throughout the experiment, pictures of wounds were captured using a digital camera. On days 0, 3, 7, 14, 21, and 28 of the experiment, the diameter of each wound was measured for each animal in the tested groups using a ruler. Moreover, the wound contraction rate (%) for each animal/group was calculated by the following equation:

$$\text{Wound}\;\text{contraction}\;\text{rate}=100*\left[\text{initial}\;\text{wound}\;\text{area}–\left(\text{area}\;\text{of}\;\text{the}\;\text{wound}\;\text{at}\;\text{given}\;\text{day}/\text{initial}\;\text{wound}\;\text{area}\right)\right].$$

#### 2.3.5. Biochemical analyses

*2*.*3*.*5*.*1*. *Preparation of tissue homogenate*. Skin samples that had been collected were rinsed with ice-cooled saline, thoroughly blotted between filter sheets, and weighed. After preparing 10% of the homogenates in ice-cooled phosphate-buffered saline (PBS, 50 mM potassium phosphate, pH 7.4), the mixture was centrifuged at 10,000 rpm for 20 min at 4°C. The supernatant was collected to measure malondialdehyde (MDA) and the total antioxidant capacity (TAC). The remaining samples of supernatant were kept at -80°C for further biochemical examinations.

*2*.*3*.*5*.*2*. *Identification of oxidative stress biomarkers*. Malondialdehyde is a lipid peroxidation product that was measured in skin tissue homogenate using the thiobarbituric acid reactive substances assay [31]. The level of TAC as an antioxidant biomarker was measured using the methodology reported by Koracevic et al. [32].

*2*.*3*.*5*.*3*. *Quantitative real-time RT-PCR analysis*. A real-time polymerase chain reaction (RT-PCR) was employed to quantify the expression of the iNOS, tumor necrosis factor-α (TNF-α), interleukin-1β (IL-1β), matrix metalloproteinase-9 (MMP-9), CD68 and CD163 genes. The QIAamp RNeasy Mini kit (Qiagen, Germany, GmbH) was used to isolate the total RNA from skin samples following the manufacturer’s instructions. The cDNA was synthesized using the HiSenScriptTM RH (-) cDNA Synthesis Kit (iNtRON Biotechnology Co., Seongnam, South Korea). The CFX96 real-time PCR detection system (CFX96; Bio-Rad, Hercules, CA, USA) was used to complete the qRT-PCR utilizing SYBR Green with low ROX TOPreal^™^ qPCR 2X PreMIX (Enzynomics, South Korea) in compliance with the manufacturer’s procedures. A preliminary denaturation at 95 ◦C for 15 min was followed by 40 cycles of denaturation at 95 ◦C for 30 s, annealing at 60 ◦C for 60 s, and a final elongation at 72 ◦C for 60 s in the PCR cycling conditions. Table 2 lists the exact oligonucleotide primers that were used in this trial. The cycle of threshold (Ct) values was quantified, and the amplification of the gene transcripts was analyzed using the ^ΔΔ^Ct method [33], employing β actin as a housekeeping gene. The control group’s gene expression level was set to 1.

**Table 2:** Primer sequences of target genes.

Gene	Direction (5′- 3′)	Primers sequences	Reference
β- actin	Forward	ATCAGCAAGCAGGAGTATGAC	(Zhang et al., 2019)
	Reverse	GCCAATCTCGTCTCGTTTCT	
iNOS	Forward	CAGGACCACACCCCCTCGGA	(Boiti et al., 2002)
	Reverse	AGCCACATCCCGAGCCATGC	
TNF-α	Forward	GTCTTCCTCTCTCACGCACC	(Godornes et al., 2007)
	Reverse	TGGGCTAGAGGCTTGTCACT	
IL-1 β	Forward	CTTTGGTTTGTTCCTGC	(Pronost et al., 1995)
	Reverse	TCATAGGTCTTTCCATCTGAA	
MMP-9	Forward	ACC TGG TTC AAC TCA CTC CG	(Tsai et al., 2008)
	Reverse	AAG ATG CTG CTG TTC AGCG	
CD68	Forward	GGTGCTGTCCTGGCTGTGT	(De Vries et al., 1999)
	Reverse	CACAGCCAGATTGAGAACT	
CD163	Forward	TACAATGGAGCTTGGGGCAG	(Jiang et al., 2020)
	Reverse	TGCCTCGATGGTGTCTTGTC	

#### 2.3.6. Histopathological and histochemical investigations

On days 7, 14, and 21, tissue specimens were collected from the wound, fixed in 10 vol. % neutral buffered formalin solution, washed, dehydrated, cleared, and embedded in paraffin. Then sectioned at 5-micron thickness and stained with Hematoxylin and Eosin for histopathological examination; also, Masson trichrome stain (MTC) was used to evaluate the collagen fiber [34]. Stained sections were examined by a light microscope (Olympus BX50, Japan).

*2*.*3*.*6*.*1*. *Histopathological and histochemical lesion scoring*. Wound healing criteria were evaluated as stated in previous work reported by [35]. Re-epithelialization was assigned a number from 0 to 4. The degree of organization was described using a 0–4 grading system for granulation tissue production. The decrease in the quantity of inflammatory cells was expressed as a degree of inflammation, ranging from 0 to 4. MTC stain was evaluated and quantified as an area % according to Baraka et al. [36] by image J 1.52 p software (Wayne Rasband, National Institutes of Health, USA).

*2*.*3*.*6*.*2*. *Immunohistochemistry assay*. The procedures for immunohistochemical analysis were as outlined by [37, 38]. Sections of the tissues were rehydrated in graded alcohol after being deparaffinized in xylene. The endogenous peroxidase activity was inhibited using a Hydrogen Peroxide Block (Thermo Scientific, USA). Tissue sections were pretreated with 10 mM citrate solution and heated in a microwave oven for 10 minutes to retrieve antigens. The following primary antibodies were incubated for two hours with the sections: anti-vascular endothelial growth factor antibody (VEGF) [MM0008-7B43] at a concentration ratio of 1: 100 (ab51867; Abcam, Cambridge, UK) and anti-transforming growth factor beta 1 antibody (TGF-β1) [EPR21143] at a concentration ratio of 1: 500 (ab215715; Abcam, Cambridge, UK). Following a PBS rinse, the sections were incubated for 10 minutes with Goat IgG H & L (HRP) (ab205718; Abcam, Cambridge, UK). The sections were washed with PBS again. Ultimately, 3, 3’-diaminobenzidine tetrahydrochloride (DAB, Sigma) was incubated in sections. After applying a hematoxylin counterstain, the slides were mounted. For the negative controls, PBS was used in place of the primary antibodies.

*2*.*3*.*6*.*3*. *Assessment of TGF-β1 and VEGF immunostaining*. Five sections of each skin group were analyzed for TGF-β1 and VEGF quantitative immunoreactivity according to previous studies [39, 40]. Under a high-power microscopic field (x 400), immunoreactivity was examined in ten microscopical areas for each section. Using color deconvolution image J 1.52 p software (Wayne Rasband, National Institutes of Health, USA), the percentage of positively stained cells (%) was calculated.

### 2.4. Statistical analysis

The findings are shown as mean ± SD or SE, and the data’s normality was determined at p > 0.05 using the Shapiro-Wilk’s test [41]. Next, one-way analysis of variance (ANOVA) and Tukey’s post hoc test were used to examine the data using the Statistical Package for Social Science (SPSS, version 17.0, Chicago, IL, USA) to determine whether there were any differences in means at p < 0.05. Graphs were prepared by GraphPad Prism 5.

## 3. Results

### 3.1. Characterization of TAD-loaded proniosomal gel

The TAD-loaded proniosomal gel was successfully prepared using different surfactants and a constant amount of cholesterol (50 mg) to study the influence of different surfactants on the characterization of the formulations. All the proniosomal gel formulations exhibited adequate viscosity resembling that of a gel. It is noteworthy that the pH values of all the gels ranged from 5.6 ± 0.02 to 6.3 ± 0.04, as presented in Table 3. These pH values fall within the normal pH range of the skin, suggesting the absence of any potential skin irritation [21].

**Table 3:** Physiochemical characterization of formulations (Mean ± SD).

Formulation code	EE, %	Size	Zeta	PDI	Viscosity	pH
F1	95.4±1.3	25.5±3.8	30.8±1.2	0.22±0.05	4120±5.9	5.6±0.02
F2	93.2±1.1	33.6±2.1	29.6±1.4	0.41±0.04	3980±7.9	5.8±0.02
F3	88.8±0.7	42.6±1.4	32.8±2.3	0.31±0.07	4020±8.2	5.6±0.02
F4	84.2±1.2	35.8±3.1	31.4±1.8	0.56±0.12	3970±4.9	5.9±0.02
F5	75.2±2.5	55.6±2.1	42.6±2.1	0.52±0.12	3955±7.4	6.2±0.02
F6	69.2±1.5	60.8±3.5	55.2±1.9	0.65±0.08	3820±6.2	6.3±0.02

#### 3.1.1. Characterization of particle size and zeta potential of TAD-loaded proniosomal gels

**Fig 1:**
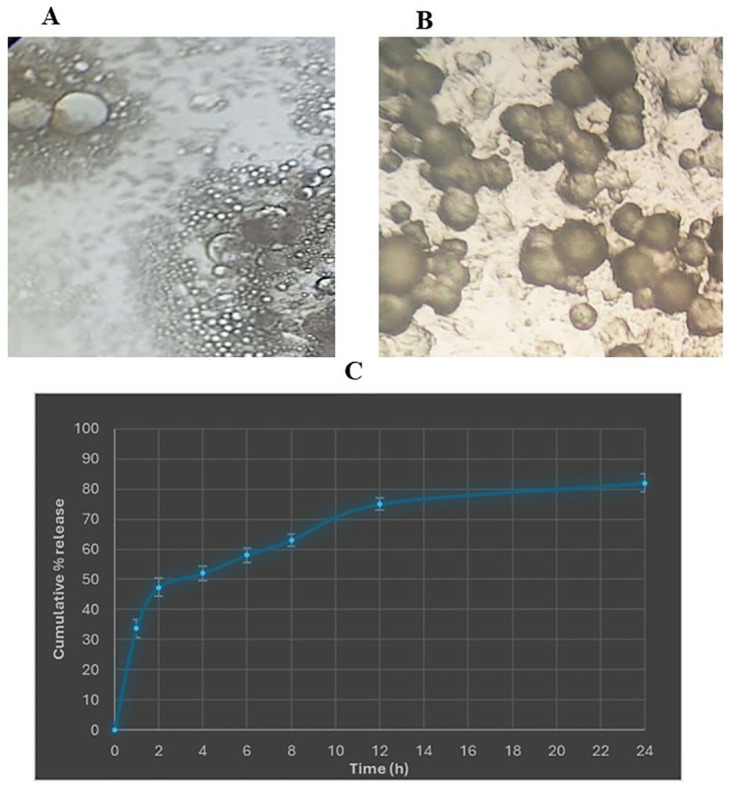
Optical photography (A), Transmission electron microscopy (B), and release profile of the chosen formula (C).

The size of all the prepared pronisomal gel particles ranged from 25.5 to 60.8 nm. The vesicles of the selected formula (F1) were distinguishable and not clustered together, as shown in Fig 1. Interestingly, the vesicles appeared smaller when span 60 was present in comparison to using Tween 80. These results could show that as the HLB value decreases, the size of the vesicles also decreases [42]. The decrease in vesicle size follows the pattern of tween 80 > span 20 > span 60. In other words, a higher HLB value leads to a reduction in surface free energy, allowing for the formation of larger vesicles with a smaller area exposed to the dissolution medium. Another explanation by [43] who have reported that spans are more hydrophobic, they form smaller vesicles to decrease their surface free energy, while tween 80 is more hydrophilic; hence, the water intake of these bilayers increased and resulted in larger niosomes. The ZP results showed good physical stability of TAD within the prepared proniosomal gels so that the aggregation of the particles was prevented and electrical stability of the system was provided [44].

#### 3.1.2. Entrapment efficiency

All formulations exhibited a high percentage of entrapment (Table 3). Proniosomes prepared using Span 20 and Span 60 had a higher level of entrapment in comparison to Tween 80-based niosomes. It was observed that formulations containing span 60 alone demonstrated higher entrapment efficiency than formulations containing span 20 and tween 80 due to their higher alkyl chain length [20]. The formulation containing only tween 80 demonstrated the lowest entrapment efficiency and this could be explained on the basis that the hydration temperature used to make niosomes should usually be higher than the system’s gel-to-liquid phase transition temperature, resulting in niosomes that are less leaky and have high entrapment efficiency. When compared to span 20 and tween 80, span 60 has the highest phase transition temperature (50°C) and entrapment efficiency%. Also, span 60 has the longest saturated chain length and the most entrapment. Both Optical photography and Transmission electron microscopy of the selected proniosomal gels at 37 °C has proved the formation of discrete niosomal vesicles.

The release profile of the chosen formula (F1) indicated that the proniosomes vesicular system effectively extended the release of TAD for 24 hours. The studies on release further revealed that a certain amount of the drug might adhere to the surface, resulting in an initial burst release of TAD followed by a gradual release. Thakur et al. obviated that the delayed release of proniosomal gel formulations is due to the slow release of drug from proniosomes and this may be attributed to the need for proniosomes for time to be hydrated to form niosomal vesicles before starting. This prolonged release over an extended period could potentially enhance patient compliance by reducing the frequency of application [24].

### 3.2. Stability studies

Stability investigations were performed on the selected formula. The proniosomal gel was kept at 4 °C for three successive months to monitor the alterations in %EE, PS, and ZP. Following storage, the EE%, PS, and ZP were 93.8 ± 1.6, 24.6 ± 1.1, and -28.4 ± 0.2, respectively.

### 3.3. In vivo study

#### 3.3.1. Gross evaluation and changes in wound diameter

**Fig 2:**
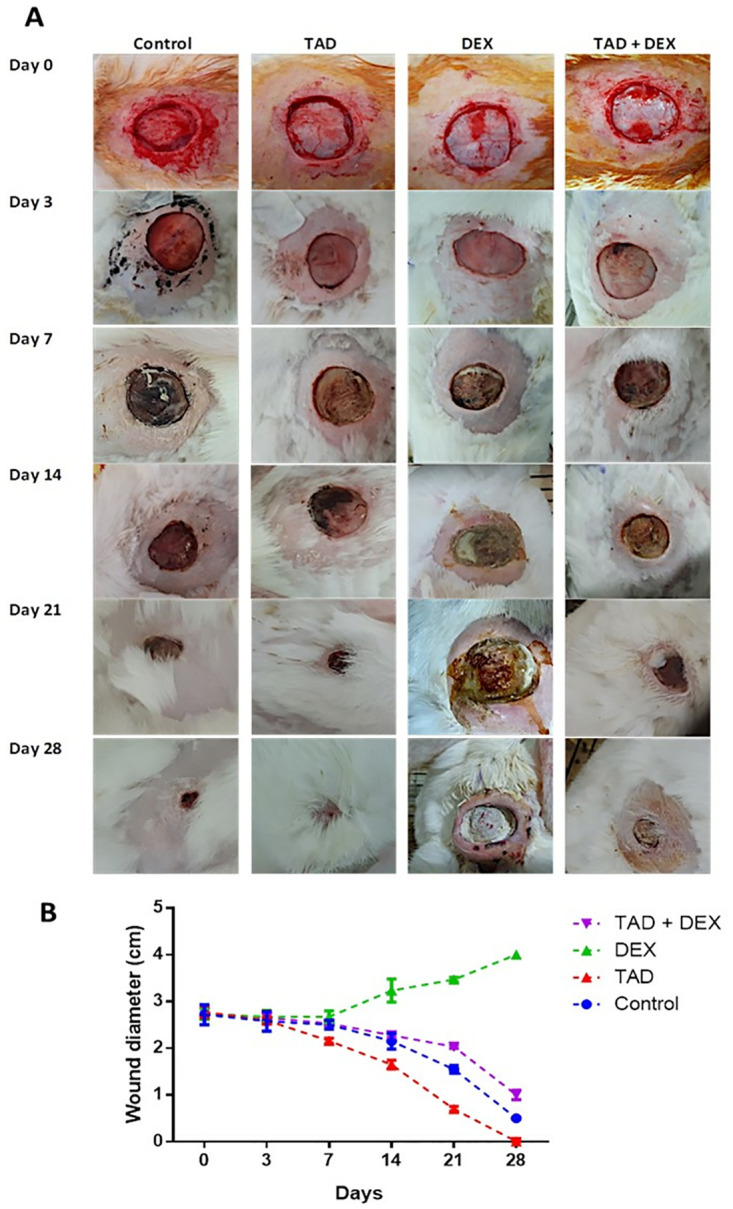
(A) Gross examination of wound area of the experimental groups, and (B) Wound diameter during the experiment period for the experimental groups. Data are presented as mean ± SD.

Fig 2A and 2B represented the wound morphology and wound healing rate of the different treated groups. At the early phase (day 3 post wounding), the healing process began in the control, TAD, and TAD + DEX groups recording a significant contraction rate (%) (p < 0.05) relative to their initials. By contrast, an inconsiderable wound closure rate was noticed in DEX-challenged rabbits accounting for 0.052% ± 0.02. By day 7 post-wounding, the healing rate % was 9.79 ± 1.40, 19.87 ± 0.66, and 9.13 ± 1.20 for the control, TAD, and TAD + DEX groups, respectively. A persistent retardation in wound healing was observed in the animals treated by DEX. In the middle stage after wounding (day 14), considerable progress in the healing process was documented in the control, TAD, and TAD + DEX groups with a wound closure rate (%) of 24.89 ± 1.32, 44.16 ± 1.34, and 20.80 ± 0.87, respectively (p < 0.05). Interestingly, inverted records of wound contraction rate were observed in DEX-challenged rabbits from the 14^th^ day post-wounding till the end of the experiment. These observations were explained by the developed pus in the DEX-treated rabbits. Conversely, the rabbits treated with TAD along with DEX administration significantly improved the wound healing process accounting for a wound contraction rate of 41.87% ± 1.30 and 61.71% ± 0.63 on day 21 and day 28, respectively. In addition, the percentage of wound closure rate at day 21 and day 28 was 51.15 ± 0.93 and 82.04 ± 0.95 for the rabbits of the control group. Likewise, on day 21 post wounding, the wound contraction rate (%) of TAD-treated animals was 76.02 ± 2.90. By day 28, a complete wound closure along with successful hair regrowth was found in TAD treated rabbits. It is worth mentioning that the kinetics of the healing process was highly accelerated in the group of rabbits treated topically with TAD-loaded proniosomal gel alone in comparison to the control and other groups. Additionally, topical application of TAD-loaded proniosomal gel along with DEX administration significantly enhanced the delayed action of DEX on wound healing.

#### 3.3.2. Biochemical studies

*3*.*3*.*2*.*1*. *Effect of topical application of TAD-loaded proniosomal gel on antioxidant/oxidant indicators*. A significant reduction in TAC along with marked boosting in MDA content was documented time-dependently in the wound tissue of DEX-treated rabbits, in comparison to the untreated animals (Table 4). On the other side, concomitant treatment with TAD-loaded proniosomal gel in combination with intramuscular injections of DEX resulted in a significant increase in tissue TAC (35.00%, 61.90%, and 85.32%, respectively) and a remarkable decline in tissue MDA level (11.75%, 27.53% and 51.21%, respectively) on days 7, 14 and 21 post-wounding compared to the DEX group (p < 0.05). It is noted that topical application of TAD-loaded proniosomal gel per se significantly elevated TAC and markedly lessened MDA content by time course interactions in wound tissue in comparison to the control group of rabbits as depicted in Table 4.

**Table 4:** Changes in total antioxidant capacity (TAC) and malondialdehyde (MDA) in the tested groups.

Groups	TAC (μmol/g tissue)			MDA (nmol/g tissue)		
	Day 7	Day 14	Day 21	Day 7	Day 14	Day 21
Control	30.05 ± 0.58 ^ab^	29.00 ± 0.61 ^b^	29.40 ± 0.40 ^b^	4.87 ± 0.09 ^c^	4.33 ± 0.09 ^c^	3.63 ± 0.20 ^c^
TAD	31.40 ± 0.36 ^a^	33.26 ± 0.42 ^a^	34.03 ± 0.92 ^a^	4.1 ± 0.17 ^d^	3.53 ±0.20 ^d^	2.39 ± 0.12 ^d^
DEX	20.57 ± 0.75 ^c^	17.40 ± 0.31 ^c^	15.33 ± 0.34 ^c^	7.83 ± 0.09 ^a^	8.10 ± 0.06 ^a^	10.31 ± 0.37 ^a^
TAD+DEX	27.77 ± 0.31 ^b^	28.17 ± 0.63 ^b^	28.41 ± 0.36 ^b^	6.91 ± 0.07 ^b^	5.87 ± 0.26 ^b^	5.03 ± 0.18 ^b^

Data are expressed as mean ± SE (n = 4). Means with distinct superscripts in the same column are significantly dissimilar at p < 0.05.

**Fig 3:**
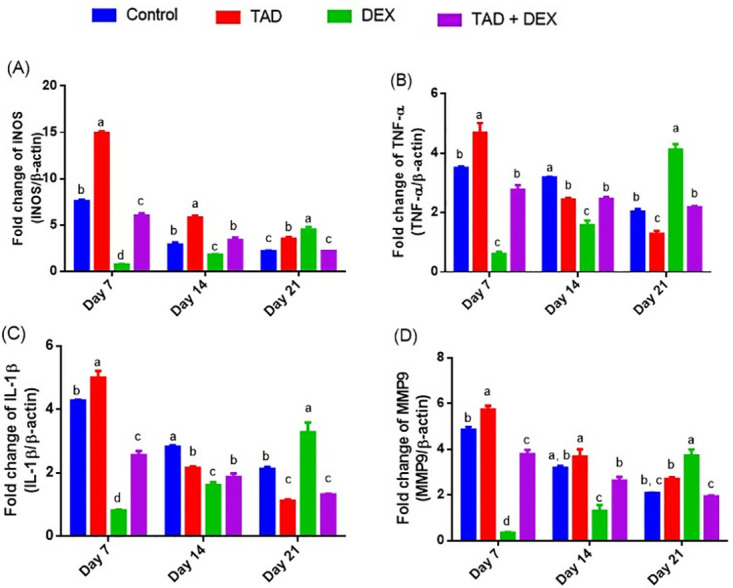
Changes in mRNA expression of iNOS (A) TNF-α (B) IL-1β (C), and MMP-9 (D) genes in the different groups. Data are expressed as mean ± SE (n = 4). Means with distinct letters in the same column are significantly dissimilar at p < 0.05.

*3*.*3*.*2*.*2*. *Effect of topical application of TAD-loaded proniosomal gel on expression of iNOS*, *TNF-α*, *IL-1β*, *and MMP-9 genes*. At the early phase post wounding/throughout healing progression, overlapping mechanisms such as the release of pro-inflammatory markers/cytokines, NO production, and extracellular matrix remodeling are collaborated as essential contributors in initiating the healing process. Intramuscular injections of DEX to the injured rabbits significantly diminished expression of iNOS, TNF-α, IL-1β, and MMP-9 genes in wound tissue on days 7 and 14, reflecting the observed delay in the wound healing process, compared to the control group (Fig 3A–3D). Additionally, on day 21, a marked elevation in the aforementioned genes in DEX-challenged animals was observed which participated in defective wound healing and chronic unhealed wound incidence, compared to the untreated rabbits. By contrast, topical application of TAD-loaded proniosomal gel to DEX-injected animals significantly regulated the release of these pro-inflammatory related genes during the different healing stages manifesting marked enhancement/progress in wound closure rate, compared to the group treated by DEX. Moreover, outstanding modulation of iNOS, TNF-α, IL-1β, and MMP-9 genes expression in wound tissue time-dependently was recorded in rabbits treated with TAD-loaded proniosomal gel only, is comparable to the control group as presented in Fig 3A–3D.

**Fig 4:**
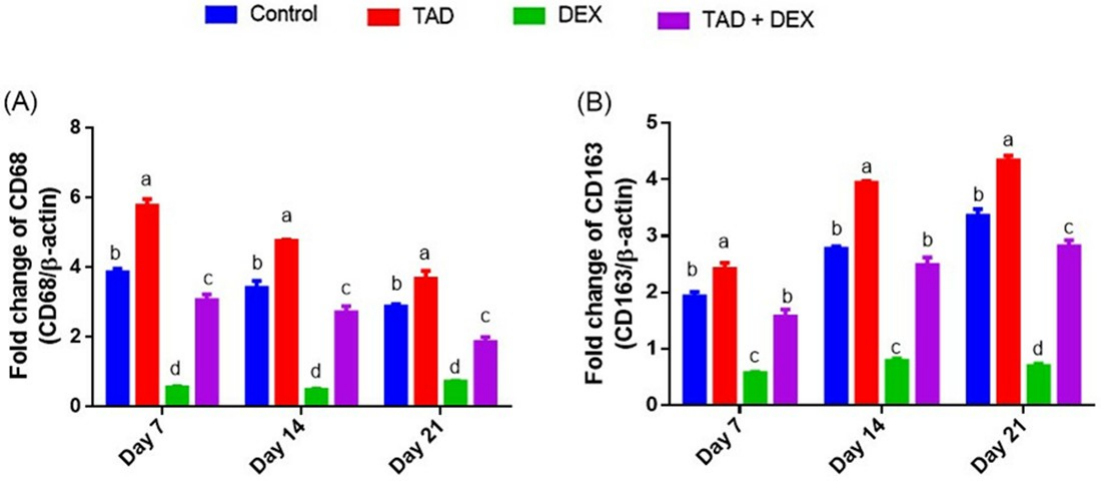
Changes in expression of macrophage activation-related genes, CD68 (A) and CD163 (B) in the experimental groups. Data are expressed as mean ± SE (n = 4). Means with distinct letters in the same column are significantly dissimilar at p<0.05.

*3*.*3*.*2*.*3*. *Effect of topical application of proniosomal gel-loaded TAD on macrophage activation-related genes*. Post wounding, activation of variable phenotypes of macrophage is a characteristic factor during different phases of the healing process in eliminating cell debris, combating bacterial infection, and even participating in collagen deposition. Concerning CD68 and CD163 genes, the immunosuppressant action of DEX resulted in a down-regulation in these genes in wound tissue, compared to the control rabbits (Fig 4A and 4B). Conversely, the topical application of TAD-loaded proniosomal gel to DEX-treated animals partially restored the expression of CD68 and CD163 genes during the different healing stages. Compared to the control rabbits, the topically administered TAD-loaded proniosomal gel significantly regulated immune response by modulating CD68 and CD163 gene levels concerning time course interactions as shown in Fig 4A and 4B.

#### 3.3.3. Histopathological findings

**Fig 5:**
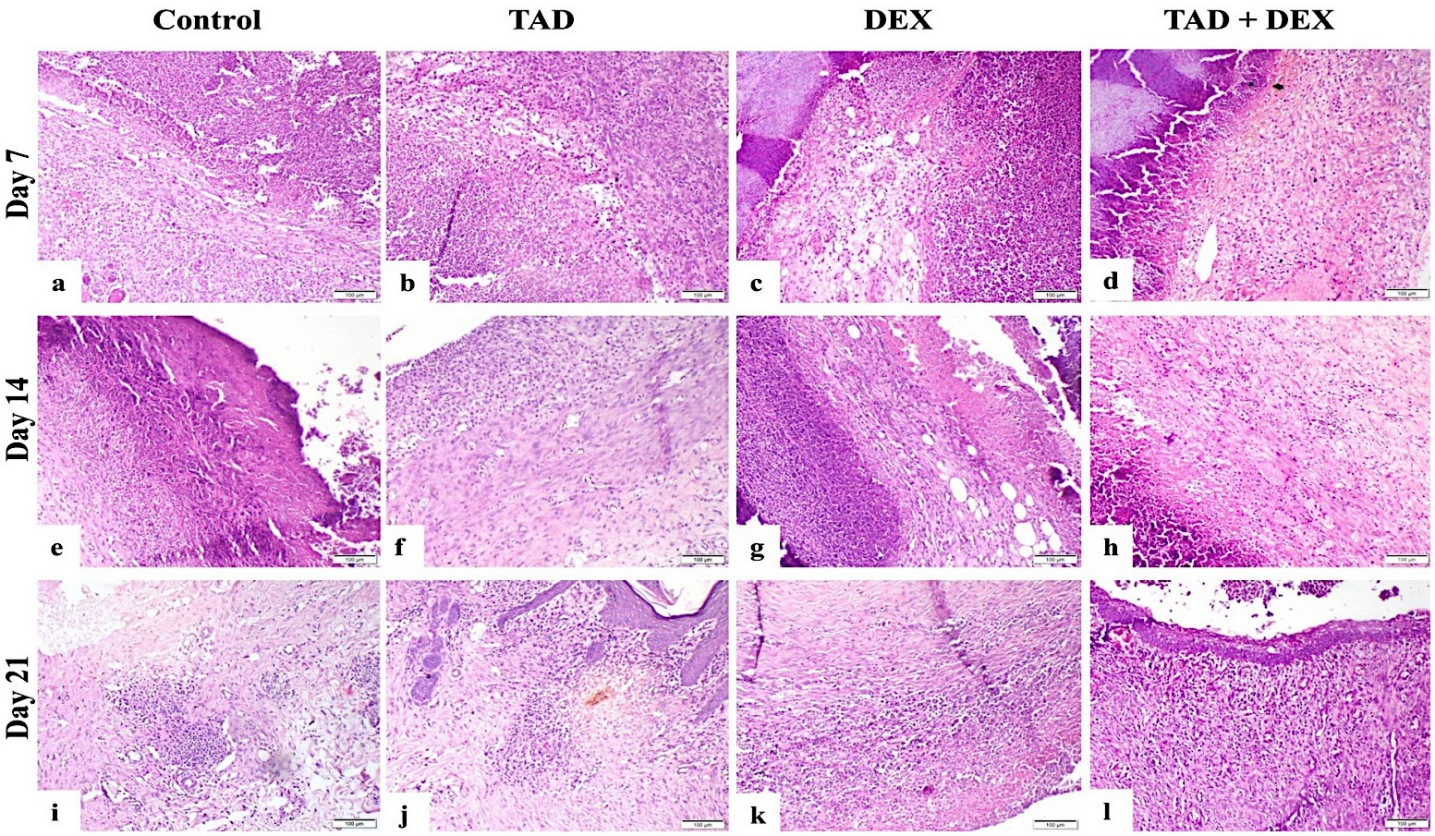
Photomicrograph of skin wound (H&E-stained sections), (a) control group on day 7 showed intense inflammatory cell infiltration and necrosis, (b) TAD group on day 7 showed intense inflammatory cell infiltration with necrosis, (c) DEX group, on day 7 showed intense inflammatory cell infiltration, edema, and scab formation, (d) TAD+DEX group at day 7 showing scab covered wound with intense inflammatory cell infiltration, (e) The control group on day 14 showed scab-covered wounds consisting of necrotic tissue and inflammatory cells, underlying tissue showing inflammatory cell infiltration, and unorganized granulation tissue, (f) TAD group on day 14 showed fewer inflammatory cell infiltration with well-arranged organized tissue, (g) DEX group, on day 14 showed intense inflammatory cell infiltration with necrosis, (h) TAD+DEX group on day 14 showed few inflammatory cells and organized tissue formation, (i) The control group on day 21 showed moderate inflammatory cell infiltration, and unorganized granulation tissue with no evidence of re-epithelialization, (j) The TAD group on day 21 showed re-epithelialization with scarce inflammatory cell infiltration and well-formed granulation tissue, (k) DEX group, on day 21 showed moderate inflammatory cell infiltration with unorganized granulation tissue, and (l) TAD+DEX group at day 21 showing mild inflammatory cell infiltration, and re-epithelialization with granulation tissue formation. (scale bar 100 μm).

*3*.*3*.*3*.*1*. *Haematoxylin and eosin stain*. Histopathological findings of the experimental animals are shown in Fig 5. Concerning the control group on day 7, there was intense inflammatory cell infiltration with the presence of necrotic tissue and hemorrhage, on day 14, this group showed scab covered wound which consisted of necrotic tissue and inflammatory cells, underlying tissue showed inflammatory cell infiltration, haphazard granulation tissue with few angiogenesis, on day 21, fewer inflammatory cells infiltration was demonstrated, unorganized granulation tissue was present with evidence of re-epithelialization. TAD group at day 7 showed intense inflammatory cell infiltration with necrosis and scab formation, at day 14, re-epithelialization started under the scab with fewer inflammatory cell infiltration, and well-arranged organized tissue was formed, at day 21, re-epithelialization was completed with scarce inflammatory cells infiltration and well-formed granulation tissue was present. Concerning the group treated with DEX, on days 7 and 14, there was intense inflammatory cell infiltration, with necrosis, hemorrhage, and fibrin exudate, at day 21, less-formed organized tissue was evident. Concerning the TAD+DEX group, on day 7, a scab covered the wound with intense inflammatory cells, and hemorrhage, necrosis, and fibrin were evident, at day 14 inflammatory cells were less observed, organized tissue formation and re-epithelialization started. on day 21, inflammatory cells were fewer, and re-epithelialization was evident with organized tissue and angiogenesis.

*3*.*3*.*3*.*1*.*1*. *Histopathological lesion scoring*: Table 5 presented the histopathological lesion score of the experimental groups as follows: re-epithelialization and formation of granulation tissue assigned as numbers ranging from 0 to 4. To describe the reduction in the number of inflammatory cells, inflammation was graded on a scale of 0 to 4.

**Table 5:** Wound healing criteria of the experimental groups.

Items	Control	TAD	DEX	TAD + DEX
*Re-epithelialization score*				
Day 7	0	0	0	0
Day 14	0	2	0	1
Day 21	2	4	0	2
*Granulation tissue formation*				
Day 7	1	1	1	0
Day 14	2	3	1	1
Day 21	2	4	2	3
*Inflammation score*				
Day 7	0	0	0	0
Day 14	1	2	0	1
Day 21	3	4	1	2

**Fig 6:**
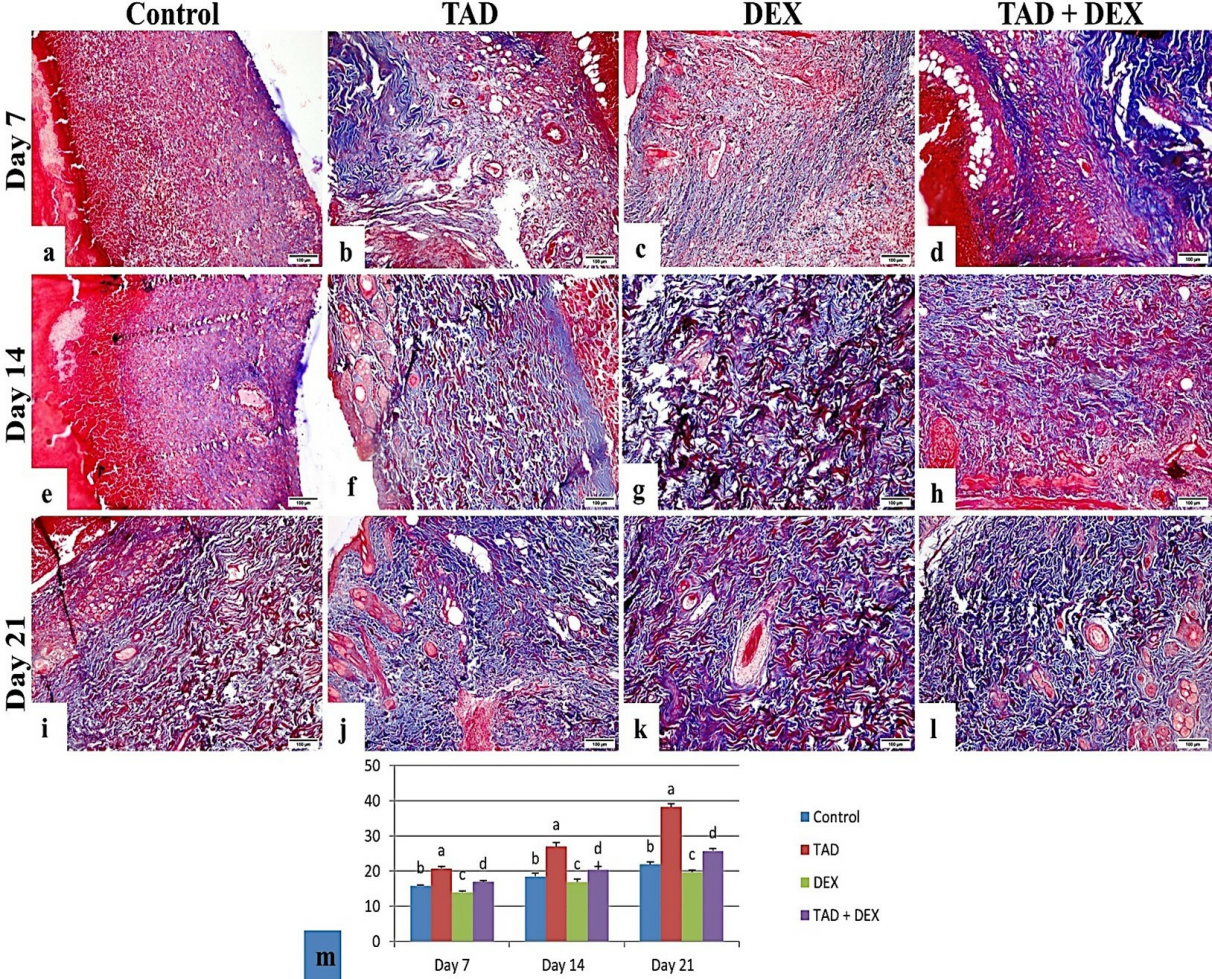
Photomicrograph of skin wound (MTC stained sections), (a) control group at day 7 showed few collagen fibers in the wound area. (b) The TAD group on day 7 showed a moderate amount of collagen. (c) DEX group, on day 7 showed few collagen fibers. (d) the TAD+DEX group on day 7 showed few collagen fibers formation. (e) The control group on day 14 showed few collagen fibers in the wound area. (f) The TAD group on day 14 showed an increase in the amount of collagen fiber. (g) DEX group, on day 14 showed disorganized collagen fibers. (h) TAD+DEX group on day 14 showed increased collagen formation. (i) The control group on day 21 showed moderate disoriented collagen fiber. (j) The TAD group on day 21 showed a high amount of well-organized collagen fiber. (k) DEX group, on day 21 showing disorganized collagen fibers. (l) The TAD+DEX group on day 21 showed moderate collagen fiber amount. (scale bar 100 μm). (m) area % of collagen fiber in different treated groups (data was expressed as mean ± SE, different letters indicating significant differences at p < 0.05). TAD; Tadalafil-Loaded Proniosomal Gel, DEX; Dexamethasone.

*3*.*3*.*3*.*2*. *Histochemical findings*. Fig 6 showed MTC stained tissue sections of different groups and area % of MTC to evaluate collagen fiber in organized tissue, the control group on days 7 and 14 showed few collagen fibers in the wound area which increased by day 21, and the TAD group showed a moderate amount of collagen at day 7 which increased significantly by day 14 and 21 respectively. DEX group showed little collagen fiber on days 7, 14, and 21 and exhibited disorganized collagen fibers, The TAD+DEX group showed little collagen fiber formation on day 7 which increased by days 14 and 21, respectively.

**Fig 7:**
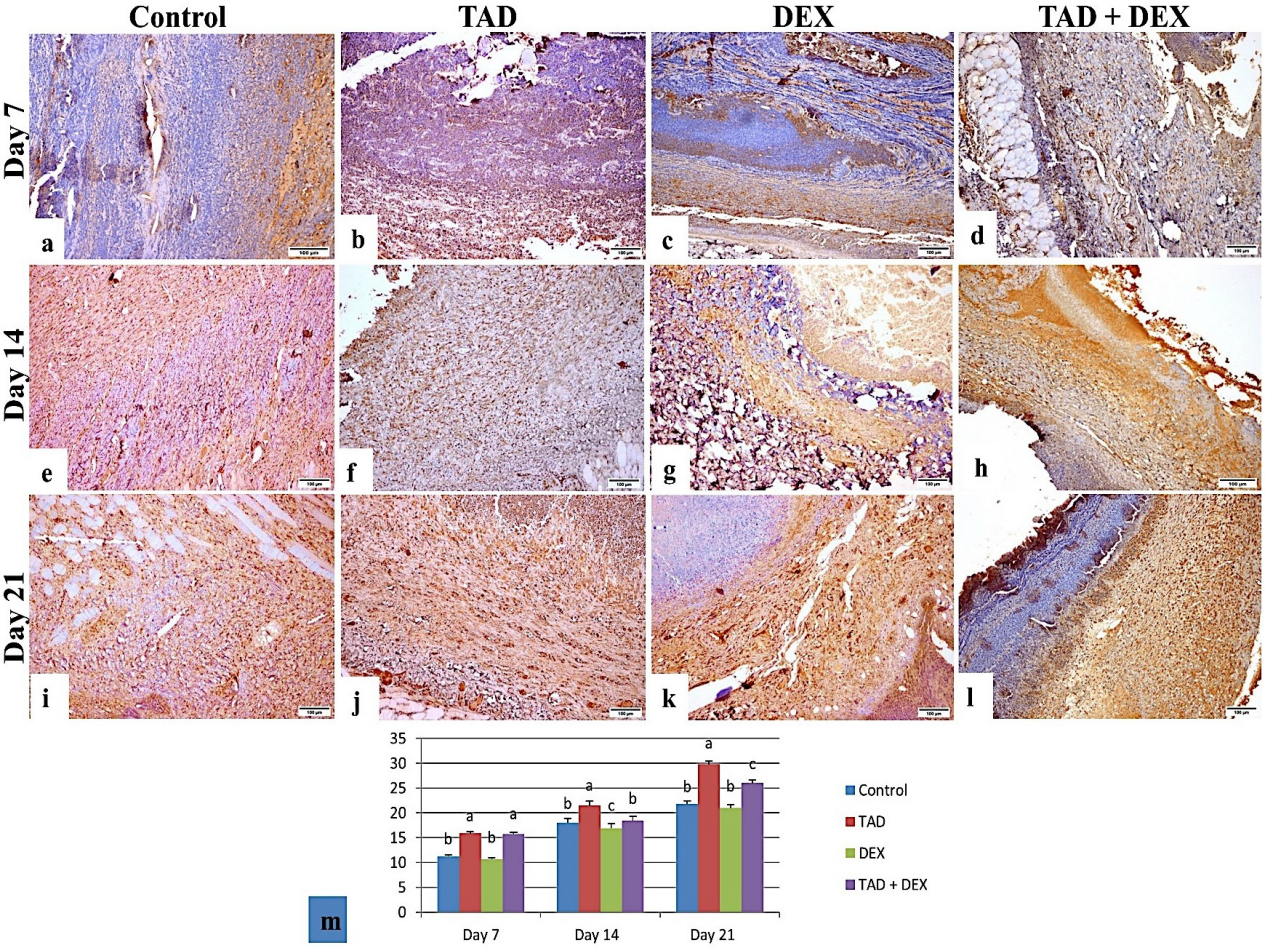
Photomicrograph of skin wound (TGF-β_1_ stained sections), (a) control group at day 7 showing weak immune expression. (b) The TAD group on day 7 showed moderate immune expression. (c) DEX group, on day 7 showed weak immune expression. (d) TAD+DEX group on day 7 showed weak immune expression. (e) The control group on day 14 showed moderate immune expression. (f) The TAD group on day 14 showed moderate immune expression. (g) DEX group, on day 14 showed moderate immune expression. (h) TAD+DEX group on day 14 showed moderate immune expression. (i) Control group at day 21 showing moderate immune expression (j) TAD group at day 21 showing strong immune expression. (k) DEX group, on day 21 showed moderate immune expression. (l) the TAD+DEX group on day 21 showed strong immune expression. (scale bar 100 μm). (m) area % of TGF-β_1_ expression in different treated groups (data was expressed as mean ± SE, different letters indicating significant differences at p < 0.05). TAD; Tadalafil-Loaded Proniosomal Gel, DEX; Dexamethasone.

**Fig 8:**
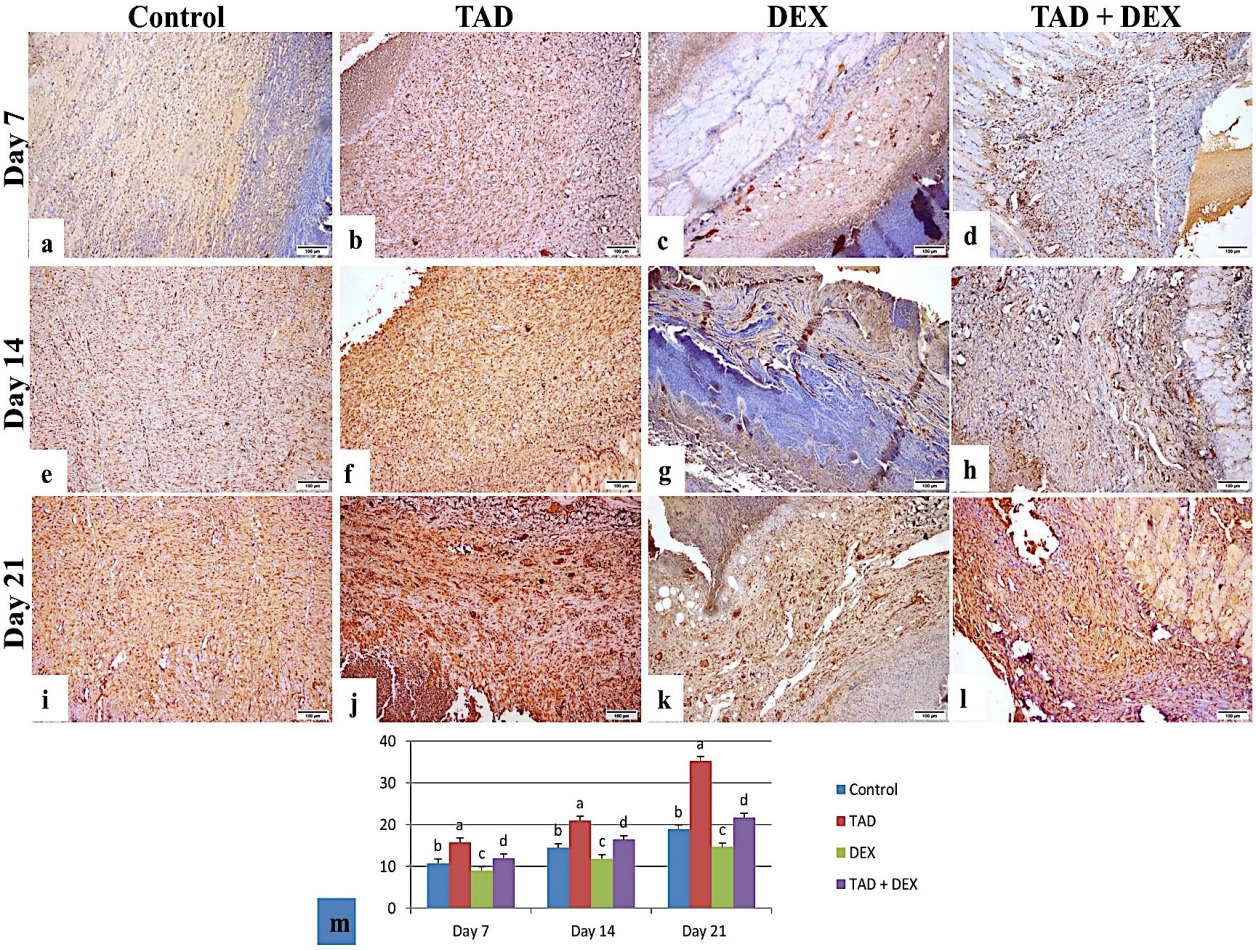
Photomicrograph of skin wound (VEGF-stained sections). (a) the control group on day 7 showed weak immune expression. (b) The TAD group at day 7 showed moderate immune expression. (c) DEX group, on day 7 showed weak immune expression. (d) TAD+DEX group on day 7 showed weak immune expression. (e) The control group on day 14 showed moderate immune expression. (f) The TAD group on day 14 showed moderate immune expression. (g) DEX group, on day 14 showed moderate immune expression. (h) TAD+DEX group on day 14 showed moderate immune expression. (i) the control group on day 21 showed moderate immune expression (j) the TAD group on day 21 showed strong immune expression. (k) DEX group, on day 21 showing moderate immune expression. (l) TAD+DEX group on day 21 showing strong immune expression. (scale bar 100 μm). (m) area % of VEGF expression in different treated groups (data was expressed as mean ± SE, different letters indicating significant differences at p < 0.05). TAD; Tadalafil-Loaded Proniosomal Gel, DEX; Dexamethasone.

*3*.*3*.*3*.*3*. *Immunohistochemistry*. Figs 7 and 8 showed immuno-reactivity and area % of TGF-β1 and VEGF respectively. Concerning the control and DEX groups, immune expression of both markers was weak on day 7 with moderate expression on days 14 and 21. The TAD group showed mild to moderate expression on days 7 and 14 and strong expression on day 21. The TAD+DEX group showed a weak expression on day 7 which increased significantly on days 14 and 21 (p < 0.05).

## 4. Discussion

Despite the presence of several formulations in the market, the cure of delayed wounds because of steroid therapy is still an obstacle for physicians [45]. Various systems have been created and loaded with different medicinal products, that boost the healing process and accelerate recovery [46]. The utilization of nanocarriers, (carrier systems for drugs) has been recently incorporated into the cure of wounds [47]. Lipid nanoparticles are a powerful carrier system for the delivery of drugs into the skin [48]. The ability of drugs to change blood flow and vascular permeability is essential for wound healing. PDE-5 inhibitors such as TAD are one example of these drugs [10]. We used a TAD-loaded proniosomal gel (delayed release), and the delayed release formulations may be linked to the need for time for proniosomal to be hydrated to noisomely vesicles before beginning. This prolonged release over an extended period could enhance patient compliance by reducing the frequency of application.

The spray-dried TDF was previously used in wound healing by [49]. Additionally, it was demonstrated that oral TDF led to quicker re-epithelialization and less scarring in a pig burn model [50]. In a rat model, oral PDE inhibitors were also shown to have a beneficial effect on the viability of skin flap repair [51]. Nevertheless, several negative and unwanted side effects, including headache, myalgia, and flushing, are brought on by oral TDF delivery [52]. Considering this, topical TDF administration may be a superior method of managing wounds.

The current study addressed the probable accelerative benefits of TAD-loaded proniosomal gel in the wound healing of rabbits treated with DEX. Impairment in the wound closure rate was recorded in DEX-administered rabbits throughout the 28 days experimental period. Steroids often hinder wound healing, strength, and closure [53]. Our results are in harmony with [54] who exhibited that when steroids were used in moderate to high doses, the wound healing process was significantly slowed down, and DEX negatively impacted all steps of the process of wound healing. Also, corticosteroids are immunosuppressant, and the retardation of healing and inadequate tissue regeneration and function result from the dysregulation of the immune responses [55]. In contrast, gradual progress in the healing process was noticed in the TAD-loaded proniosomal gel + DEX group relative to the DEX treated rabbits from days 3, 7, 14, and 21 post-wounding until the end (day 28). The results of [49] are consistent with the current findings.

Additionally, Luo and Chen [56] verified that when compared with normal skin, the wound sites treated with spray-dried pro nanoliposomes loaded with tadalafil demonstrated properly formed, fully healed skin with collagen deposition, the onset of hair follicle creation, and connective tissue. The decline in cGMP breakdown and thus, the long duration of NO influence on the cellular and endovascular levels; is the cause of PDE-5 inhibitors’ wound healing impact. This elevation in NO levels has a positive impact on the wound-healing process [57]. The wound healing-enhancing action of NO happens on various levels involving angiogenesis, collagen deposition, endothelial and epithelial cell proliferation, inflammation, and remodeling [58].

Rabbits administered DEX exhibited an oxidant/antioxidant imbalance as illustrated by a remarkable decline in TAC with a marked rise in MDA time-dependently in wound tissue, versus the control group. Also, Rao et al. [59] reported significant reductions in catalase, superoxide dismutase, glutathione, and glutathione transferase in DEX-administered rats. This demonstrates that DEX stimulated some levels of oxidative damage. High levels of free radicals may increase the duration of inflammation by causing oxidative damage to the wound, neovascularization damage, and metabolic damage [60]. Important causes for hindering the healing of chronic wounds are low NO content and high reactive oxygen species (ROS) levels [61]. However, concomitant administration of TAD-loaded proniosomal gel with intramuscular injections of DEX leads to a remarkable elevation in TAC and a decline in tissue MDA on days 7, 14, and 21 post-wounding. Oxidant/antioxidant imbalance can be caused by the injury itself. Molecular oxygen plays a key role in the process by which wounds develop. When ROS are created in considerable amounts, detrimental cytotoxic impacts happen, causing a prolongation in wound healing. Therefore, to enhance wound healing, one important point is to decrease ROS production [62].

Dexamethasone-injected rats revealed delayed wound healing by significant downregulation in the gene expression of iNOS, TNF-α, IL-1β, and MMP-9 genes in wound tissue at days 7 and 14 post-wounding and a remarkable rise in these genes at day 21 relative to the control. TNF-α, IL-1β, and iNOS are inflammatory cytokines that are secreted by M1 macrophages [63] and can eliminate infectious organisms such as bacteria, viruses, and malignant tumor cells, then macrophages phagocytose cells that are dead [64]. Therefore, M1 macrophages are considered to be included in the preservation of human homeostasis by protection from infection. [8] went hand in hand with us and confirmed that steroids have an adverse influence on the healing of wounds by prolonging the inflammation stage and suppressing the proliferation stage. Steroids suppress the inflammatory stage (reduce vascular permeability, extravasation, suppress complement and lymphocyte derived factors), angiogenesis, fibroblastic proliferation, and ECM (proteins and proteoglycans) production [65]. Vasoconstriction, a changed vascular redox situation, abnormal vascular smooth muscle cell growth, and prothrombotic alterations in the vessel wall can all be brought on by low NO levels in the wound milieu, which can impair the healing of the wound [66]. Matrix metalloproteinases (MMPs) are generally needed in trace quantities and oversee appropriate epithelization and keratinocyte proliferation. MMPs are secreted by immune cells, fibroblasts, and keratinocytes in response to local mediators, which include growth factors and cytokines involved in wound healing. However, their lack of regulation is closely linked to wounds that are difficult to heal and result in poor epithelialization [67]. Also [68] revealed that various MMPs, involving MMP-2 and MMP-9, are secreted by macrophages in chronic wounds that decline the ECM remodeling and inhibit the start of the proliferative stage of healing. The cause of impediment in the repair of chronic wounds may be the high level of MMP-9 expression induced by activated neutrophils [55].

On the contrary, rabbits administered TAD-loaded proniosomal gel + DEX improved the delay in wound healing relative to DEX-injected rabbits. TAD elevated NO levels which starts a cascade of processes that accelerate the recovery of wounds, from angiogenesis to tissue remodeling [56]. The high levels of NO in the healing process for wounds have a major function in angiogenesis by its impact on the pro-angiogenic action of cytokines. Also, elevated NO levels stimulate the latent TGF-ß1 factor, which aids in the chemo-attraction of wounds. In addition, NO molecules play a direct role in recruiting monocytes [69]. Moreover, NO suppresses the inflammatory stages by preventing additional monocyte migration, allowing the healing process to transfer from the inflammatory to the regenerative stages [70]. The subsequent initiated phase of NO enhances proliferative activity by promoting endothelial cell proliferation, acting as a mediator for the synthesis of VEGF, and shielding endothelial cells from apoptosis, (a type of programmed cell death) [71].

Topically applied TAD-loaded proniosomal gel successfully modulated the expression of macrophage activation-related genes during the various stages of wound healing, CD68, and CD163, relative to the DEX group. The immunosuppressant action of DEX is directly related to the deactivation of these macrophage expression genes which clearly emphasizes the worsening progress in wound healing rate [55] and increases the possibility of wound infection, as reported in the current study. The cell-surface glycoprotein receptor CD163 serves as a distinctive marker for monocytes and macrophages that exhibit anti-inflammatory characteristics [72]. Macrophages have two characteristic polarization phases: the classically activated M1 phenotype (CCR7+) and the alternatively activated M2 phenotype (CD163+) [73]. M1 macrophages stimulate inflammation, whereas M2 macrophages have anti-inflammatory impacts and assist in different elements of wound healing [74]. A crucial step in the effective healing process is the conversion of M1 to M2 macrophage phenotypes. If this transformation fails, it can lead to persistent wounds such as diabetic wounds and venous ulcers, or it might hinder wound healing [75]. This proposes that TAD-loaded proniosomal gel enhanced the healing of wounds by aiding M1-to-M2 macrophage phenotype transformation.

Immunohistochemistry revealed a significant elevation in the immune expression of TGF-β1 and VEGF in the TAD-loaded proniosomal gel + DEX group time-dependently compared with DEX-injected rabbits. The most potent signaling protein that promotes angiogenesis, wound healing, and re-epithelialization is the inflammatory cytokine VEGF [76]. Chemokines, cytokines, and growth factors such as TGF-α, TGF-β, bFGF, PDGF, and VEGF are released by activated macrophages to intensify and eventually overcome inflammation [67]. NO has an impact on VEGF, which is important for blood vessel synthesis [77]. The latent TGF-ß1 is stimulated by high levels of NO which is linked to wound chemoattraction, furthermore, NO molecules are directly included in the recruitment of monocytes [70].

The histopathological findings documented the deteriorating effects of DEX on wounds as evidenced by the observed disorganized collagen deposition with numerous activated neutrophils and inflammatory cell infiltration which explained the retardation in the wound healing process and even the progression of pus formation in the DEX-challenged group [45]. Contrary to that, a significant improvement in pathological alterations was observed in TAD-loaded proniosomal gel-treated rabbits, concerning granulation, re-epithelization, collagen deposition, and inflammatory cells.

## 5. Conclusion

Our results emphasized the efficacy of TAD-loaded proniosomal gel in enhancing the delayed action of corticosteroid agent, DEX, on the wound healing process. Interestingly, topically applied TAD-loaded proniosomal gel manifested a superior ability to accelerate the wound closure rate by regulating inflammatory response, macrophage transformation, collagen deposition, re-epithelization, and hair regrowth.

## Abbreviations

cGMP: cyclic guanosine monophosphate
DEX: dexamethasone
ECM: extracellular matrix
H&E: Hematoxylin and eosin
IL-1β: interleukin-1β
iNOS: inducible nitric oxide
MDA: malondialdehyde
MMP-9: matrix metalloproteinase-9
MMPs: matrix metalloproteinase
MTC: Masson trichrome stain
NO: nitric oxide
PDE-5: phosphodiesterase type 5
ROS: reactive oxygen species
RT-PCR: real-time polymerase chain reaction
TAC: total antioxidant capacity
TAD: tadalafil
TEM: Transmission electron microscopy
TGF-β: transforming factor-beta
TNF-α: tumor necrosis factor α
VEGF: vascular endothelial growth factor
